# Wunderlich's syndrome and hemorrhagic shock

**DOI:** 10.4103/0974-2700.55346

**Published:** 2009

**Authors:** Massimo Medda, Stefano CM Picozzi, Giorgio Bozzini, Luca Carmignani

**Affiliations:** Cardiovascular Interventional Radiology Service, University of Milan, San Donato Milanese, Milan, Italy; 1 Urology Department, IRCCS Policlinico San Donato, University of Milan, San Donato Milanese, Milan, Italy

**Keywords:** Angiomyolipoma, embolization, kidney, massive hemorrhage, renal tumor

## Abstract

We report a case of Wunderlich's syndrome in an obese woman associated with massive retroperitoneal hemorrhage. Stable hemodynamic patient condition was obtained by selective arterial embolization. Since the first embolization of a renal angiomyolipoma in 1976 by Moorhead *et al.*, highly selective renal arterial embolization of angiomyolipoma with rupture has become a procedure that offers greater efficacy, particularly in life-threatening cases.

## INTRODUCTION

Classic angiomyolipoma is considered to be benign mixed mesenchymal tumors that occurs predominantly in the kidney. These tumors consist of a collection of thick-walled blood vessels, smooth muscle and mature adipose tissue in varying proportions. A second type of angiomyolipoma is described containing a large fourth component, the perivascular epithelioid cells, making it more aggressive than the classical form. The incidence in the general population is between 0.07 and 0.3%.[1] Approximately 80% of renal angiomyolipoma occur sporadically and 20% are associated with tuberous sclerosis. In the sporadic cases, these lesions are found usually larger, single and unilateral, with a female preponderance (approximately 4:1) in the fourth to sixth decade of life.

Several studies have demonstrated that the frequency of symptoms and the risk of bleeding increase with the size of the lesion. Approximately 64-77% of tumors < 40 mm in diameter are asymptomatic, although 82-90% of angiomyolipoma >40mm produce symptoms.[2]

In symptomatic patients, the classic presentation includes flank or abdominal pain, a palpable tender mass and gross hematuria (Lenk's triad). Other symptoms as nausea, vomiting, fever, anaemia, renal failure and hypotension are observed less frequently. Three types of hemorrhagic etiology exist, including Wunderlich's syndrome (spontaneous retroperitoneal hemorrhage of nontraumatic origin), bleeding or rupture after trauma and rupture during pregnancy (secondary to a rapid hormonal-related growth). We present a case of Wunderlich's syndrome in an obese woman associated with hemorrhagic shock.

## CASE REPORT

An obese 50-year-old Caucasian woman (body mass index 35.2) was admitted to our Emergency Department with right-sided abdominal pain of sudden onset at 4.50 pm. There was no significant past medical or family history. General examination was normal except for pallor. Her hemodynamic parameters were stable; her blood pressure was 135/80 mmHg, pulse was 96/min and O_2_ saturation was 98% in room air. There was no fever. Cholecystitis was suspected, because Murphy's sign was positive, and analgesic treatment was administered at 5.15 pm. Initial blood test revealed a hemoglobin level of 13.5 g/dl, a white blood cell count of 11300/ul, a platelet count of 256.000/ul, liver and renal function in the normal range, C-reactive protein of 9 mg/dl, normal coagulation tests and negative urinalysis. Abdomen ultrasonography was requested to the Radiological Department.

**Figure 1:**
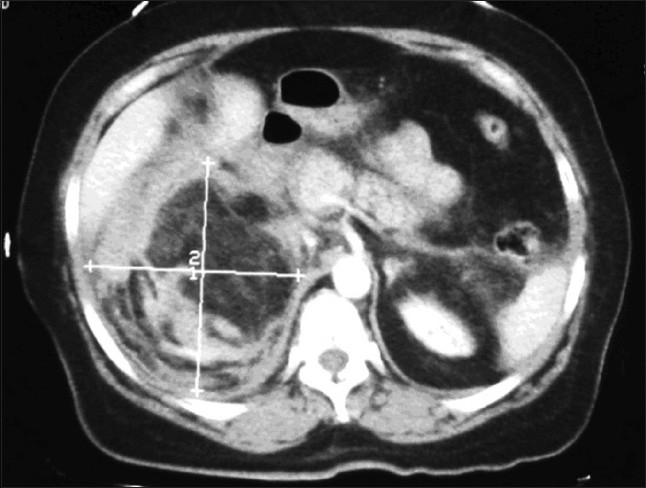
Abdominal computed tomography scan showing a 22 × 15 cm right renal fat mass with a perirenal haematoma in the right kidney

At 6.50 pm, the patient's symptoms worsened and she developed nausea, vomiting, hypotension (80/50 mmHg), tachycardia (125 bpm), confusion and diaphoresis. Her hemoglobin dropped to 8.5 g/dl. Urgent ultrasonography and computed tomography showed a 22 × 15 cm right renal fat mass associated with massive recent hemorrhage (findings suspicious of a bleeding renal angiomyolipoma) [Figure 1].

The patient's condition precipitated. Supportive therapy with ventilation, monitoring and establishment of a central venous access in addition to two large-bore catheters in peripheral lines was initiated. Fluid treatment included repeated aliquots of 250ml of Ringer's solution and 6% hydroxyethyl starch 130/0.4 in 0.9% sodium chloride injection solutions, administered with continuous monitoring, with re-establishment of a systolic blood pressure of 70 mmHg and appearance of a radial pulse.

**Figure 2:**
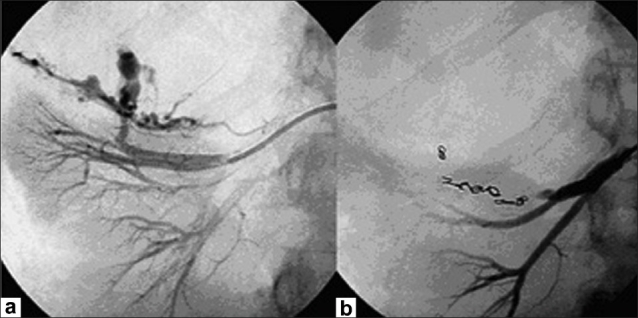
(a) Right renal artery angiogram showing the principal feeding vessel and abnormal vessels containing areas of aneurysmal dilatation supplying the angiomyolipoma; (b) Angiogram after selective embolization of the upper pole branch of the renal artery with coils, demonstrating obliteration of the vascular supply to the tumour

**Figure 3:**
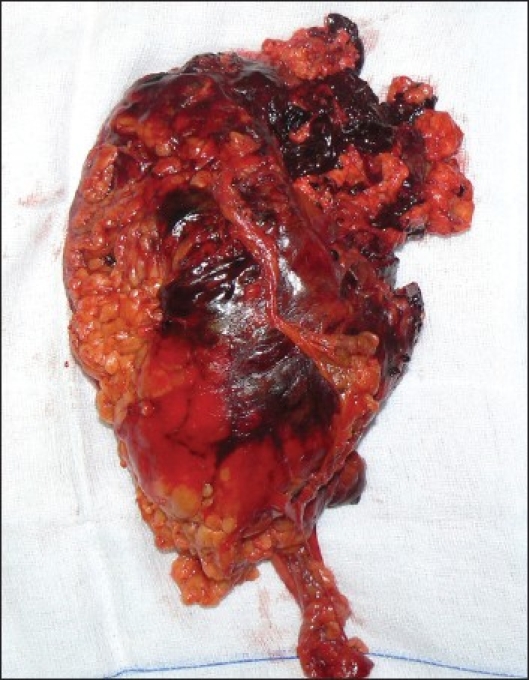
Surgically resected right kidney involved by a large fatty mass located at the upper pole with signs of haemorrhage and haematoma

**Figure 4:**
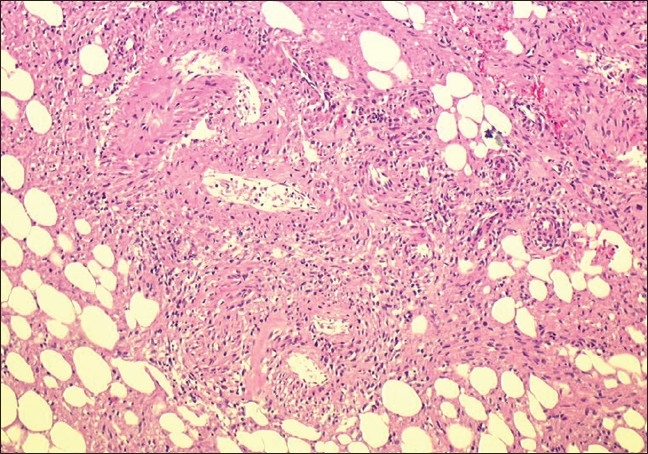
Histological section of the specimen shows that the lesion was composed of a mixture of thick walled blood vessel, fat cells, and spindle smooth muscle-like cells (H and E stain, × 10)

The decision-making process performed by the urologist resulted in alerting the Emergency Cardiovascular Interventional Radiology Service. At 7.15 pm, after resuscitation, selective embolization of the upper pole branch of the renal artery (the lesion-supplying artery) was performed with 2/20 mm and 3/20 mm coils [Figure 2]. After the procedure, the patient's hemodynamic condition was stable and she was transferred to the intensive care unit where she received a total of 8 units of packed red blood cells and 2 units of fresh frozen plasma. Following embolization, the patient's general condition improved and an elective exploratory laparotomy was performed 3 days after embolization. At surgery, a huge retroperitoneal hematoma extending to the pelvis was found. The size of the tumor was of importance and a right nephrectomy was performed [Figure 3]. The postoperative recovery was uneventful. Histology confirmed a renal angiomyolipoma [Figure 4].

## DISCUSSION

Spontaneous nontraumatic bleeding confined to the subcapsular and/or perinephric space in patients with no known underlying cause was first described as “spontaneous renal capsule apoplexy” by Carl Reinhold August Wunderlich in 1856.[3] Presentation of this clinical picture may vary greatly depending on the degree and duration of the bleeding. Commonly acute lumbo-abdominal pain, nausea, vomiting, hematuria, hemodynamic instability, anemia and hypovolemic shock are present. Several causes are involved, including benign and malignant renal neoplasms, vascular disease (vasculitis, renal artery arteriosclerosis and renal artery aneurism rupture), nephritis, infections, undiagnosed hematological disorders and anatomical lesions. Wunderlich's syndrome is one of the most feared complications of renal angiomyolipoma and can be fatal if not treated promptly and aggressively. It occurs in up to 50% of patients with tumors larger than 40 mm because of the association with an increased risk of intralesional aneurismal formation and, therefore, a greater possibility of rupture.[4] In fact, having abnormal elastin-poor vascular structures, angiomyolipomas are likely to form aneurisms as they grow and as the blood flow entering them increases.

Computerized tomography is the method of choice for the demonstration of perirenal hemorrhage (sensitivity of 100%) and, if performed during the time of hemorrhage, it has been found to identify all cases of Wunderlich's syndrome due to angiomyolipoma.[4,5]

In 1986, Oesterling *et al*. proposed a treatment protocol based on size and symptoms: asymptomatic tumors < 40 mm should be observed regularly with computerized tomography or ultrasound whereas those > 40 mm should be studied by angiography and considered for arterial embolization or surgery.[2]

Since the first embolization of a renal angiomyolipoma in 1976 by Moorhead *et al.*, highly selective renal arterial embolization of angiomyolipoma with rupture has become a procedure that offers greater efficacy in life-threatening cases.[6] The major advantages of embolization in these cases include its minimal invasiveness, the capacity of stopping acute hemorrhaging in emergencies, relieving patient symptoms, preventing further hemorrhaging, feasibility of retreatment and preservation of renal function in preventing a nephron-sparing surgery.

## CONCLUSION

A case of Wunderlich's syndrome associated with massive retroperitoneal hemorrhage is reported. Wunderlich's syndrome is one of the most feared complications of renal angiomyolipoma and can be fatal if not treated promptly and aggressively. The appropriate treatment for this condition depends on the accuracy of diagnosis and the determination of its cause. Highly selective renal arterial embolization of angiomyolipoma with rupture permits to obtain stable hemodynamic patient condition in emergency. This procedure has become a procedure that offers greater efficacy, particularly in life-threatening cases.
